# Perceived neighborhood disorder and type 2 diabetes disparities in Hispanic, Black, and White Americans

**DOI:** 10.3389/fpubh.2024.1258348

**Published:** 2024-01-15

**Authors:** Min Ying Yu, Alfredo J. Velasquez, Belinda Campos, Jennifer W. Robinette

**Affiliations:** ^1^Department of Psychology, Chapman University, Orange, CA, United States; ^2^Department of Human Development and Family Sciences, The University of Texas at Austin, Austin, TX, United States; ^3^Department of Chicano/Latino Studies, University of California at Irvine, Irvine, CA, United States; ^4^Department of Psychological Science, University of California at Irvine, Irvine, CA, United States

**Keywords:** race/ethnicity, perceived neighborhood disorder, type 2 diabetes, health disparities, neighborhood environment

## Abstract

**Introduction:**

Approximately 32 million Americans have type 2 diabetes, and that number continues to grow. Higher prevalence rates are observed among certain subgroups, including members of marginalized racial/ethnic groups as well as residents of disordered neighborhoods (i.e., those with more trash and vandalism). Institutionalized discriminatory practices have resulted in disproportionate representation of marginalized racial/ethnic groups in disordered neighborhoods compared to non-Hispanic Whites. These neighborhood disparities may partially contribute to health disparities, given that signs of neighborhood disorder often relate to a general withdrawal from the neighborhood, minimizing opportunities for both physical and social engagement. Yet, research suggests variability across racial/ethnic groups both in reporting rates of neighborhood disorder and in the extent to which neighborhood disorder is interpreted as posing a threat to health and well-being.

**Methods:**

Using 2016–2018 Health and Retirement Study data (n = 10,419, mean age = 67 years), a representative sample of older US adults, this study examined the possibility of racial/ethnic differences in associations between perceived neighborhood disorder and type 2 diabetes risk. Participants reported their perceptions of neighborhood disorder and type 2 diabetes status. Weighted logistic regression models predicted type 2 diabetes risk by perceived neighborhood disorder, race/ethnicity, and their interaction.

**Results:**

Non-Hispanic Blacks and Hispanics had higher type 2 diabetes risk; these two groups also reported more disorder in their neighborhoods compared to non-Hispanic Whites. Perceiving more neighborhood disorder was associated with increased type 2 diabetes risk, but the interaction between race/ethnicity and disorder was not significant.

**Discussion:**

Findings from the current study suggest that the negative effects of perceiving neighborhood disorder, a neighborhood-level stressor, extend to increased type 2 diabetes risk.

## 1 Introduction

Over 30 million US adults have type 2 diabetes and an additional 80 million have prediabetes (1), with experts predicting that one out of every three adults will have type 2 diabetes by 2050 (2). Type 2 diabetes is also a risk factor for comorbid conditions such as hypertension and obesity (3). Many scholars have focused on individual-level etiological factors such as lower socioeconomic status (4–6) that increase disease risk. Racial/ethnic disparities in type 2 diabetes risk also exist, in which disparities are defined as the unequal burden of diseases and other adverse health conditions that exist among certain subgroups of a population (7). Researchers find that Hispanic and non-Hispanic Black individuals are more likely to have type 2 diabetes compared to non-Hispanic Whites (8–10). According to the American Diabetes Association, type 2 diabetes is less prevalent among non-Hispanic Whites (7.5%) than Asian Americans (9.2%), Hispanics (12.5%), non-Hispanic Blacks (11.7%), and American Indians/Alaskan Natives (14.7%) (11).

Beyond individual characteristics, features of neighborhood environments are also associated with increased disease risk (12, 13). In fact, the World Health Organization states that social determinants of health are linked to “the conditions in which people grow up, live, and age” and that inequality in these spaces can lead to poorer health outcomes across generations (14). The focus of the current analyses is on perceived neighborhood disorder, or the degree to which residents feel unsafe or observe vandalism, trash in the streets, and abandoned buildings. In particular, we set out to investigate whether differential exposure to neighborhood disorder relates to a differential association between neighborhood disorder and health across White, Black, and Hispanic groups.

The neighborhood environment can serve as a health-promoting pillar supporting both health and well-being (15). Neighborhood environments may be an even stronger predictor of health in the context of older adulthood, as older adults may spend more time in their neighborhoods (16–19). Specifically, neighborhood social and physical disorder is linked to poorer health among residents (13, 20–23). Neighborhood disorder theory suggests that residents modify their behavior due to perceptions of threat toward safety and well-being (24, 25). Residents of neighborhoods perceived as disordered who fear victimization may withdraw to their own homes, reducing opportunities for making social connections and increasing sedentary behaviors in ways that increase risk of type 2 diabetes (22, 26–28). Third-party ratings of physical and social disorder incivilities have indeed been related to increased fear of victimization, a reduced likelihood of going out for leisurely-related activities, and decreased levels of physical activity (29, 30).

Residents who report lower neighborhood safety, which is considered a component of neighborhood disorder, have more chronic health conditions (31). Additionally, residents with type 2 diabetes who report greater physical and social disorder in their neighborhoods experience higher overall distress related to diabetes management (i.e., regimen-related, physician-related, emotional, interpersonal) and worse glycemic control and adherence to medical regimen than those who report less disorder in their neighborhoods (32).

In addition to disparities in disease risk, racial/ethnic disparities exist in exposure to the environmental features with demonstrated links to type 2 diabetes (10, 32, 33). Non-Whites are also less likely to move out of disordered neighborhoods, or to move into more advantaged neighborhoods due to mobility restraints (24). Furthermore, greater economic disinvestment in communities with higher proportions of Hispanics and non-Hispanic Blacks reduce resources that would otherwise enable the maintenance of healthy lifestyles (34, 35). As such, exposure to neighborhood disorder is often more characteristic of the non-White, compared to the non-Hispanic White experience, and may contribute to notable health disparities.

Beyond these observed neighborhood differences, researchers have begun realizing racial/ethnic differences in the interpretation of neighborhood disorder as a sign of personal threat (13, 24, 36). Non-Hispanic Whites report disorder more often than those from other racial/ethnic groups, for example, which some argue results from relatively low levels of exposure to disorder among non-Hispanic Whites to begin with (37). Similarly, a growing line of research indicates that differences in exposure to, and feelings of ‘insecurity’ in the context of neighborhood disorder may determine further racial/ethnic differences in interpretation of neighborhood disorder as a cue for potential victimization (38).

Very few studies have investigated racial/ethnic differences in the effect of neighborhood disorder on health or health-related outcomes. This paucity of research thwarts the ability to further understand environmental correlates of racial/ethnic health disparities. A few existing investigations have started the conversation by investigating psychosocial outcomes. The Compound Risk theory (39) suggests that non-Whites are more likely to live in neighborhoods with plentiful stressors, including signs of neighborhood disorder. These stressors were hypothesized to result in a greater depletion of mastery, or the personal sense that one can influence their own life circumstances, among non-Whites. As such, this theory suggests a stronger association between signs of disorder and poor health among non-Whites. Conversely, others observed a stronger association between neighborhood disorder and reduced personal control among non-Hispanic Whites compared to other racial/ethnic groups (40). They argued that perceiving neighborhood disorder created greater cognitive dissonance, or a mismatch between personal social status and the status of one’s neighborhood, among non-Hispanic Whites, rendering non-Hispanic Whites as more vulnerable to neighborhood disorder compared to other racial/ethnic groups. These authors coined this phenomenon the Status Discord Theory (40).

Even fewer investigations examine racial/ethnic differences in associations between neighborhood disorder and health outcomes. One recent study observed that neighborhood physical disorder as rated by third parties was related to safety concerns, but only among non-Hispanic Whites and Hispanics, not non-Hispanic Blacks. However, neighborhood physical disorder was linked to poorer self-rated health among all three groups (13). These results suggested that, despite differences in interpretation of personal threat, ameliorating signs of physical disorder may be a worthy neighborhood intervention that benefits diverse populations.

Few studies have investigated potential racial/ethnic differences in associations between perceived neighborhood disorder and health outcomes, which limits our understanding of racial/ethnic health disparities. Taken together, the purpose of the present study was to investigate potential racial/ethnic differences in perceived neighborhood disorder and type 2 diabetes using a representative sample of older US adults. This analysis was motivated by, and directly relates to one of the objectives set in Healthy People 2030, including reducing the burden of type 2 diabetes and emphasizing an ecological approach that incorporates environmental risk factors (41).

## 2 Materials and methods

### 2.1 Data

The Health and Retirement Study (HRS) is a nationally representative sample of US adults aged 51 and older. The survey is designed to examine the health and retirement status of older adults to explore the developmental complexities involved with aging (42). Beginning in 1992, HRS gathered information about participants’ economic, physical, mental, and cognitive well-being through a core interview (conducted face-to-face at baseline and by telephone during follow-up assessments) biennially. To ensure a representative sample of people aged 51 and older in the US, new cohorts have been added to the survey every 6 years. In 2006, HRS added an enhanced face-to-face interview (EFTF) on a random half of the sample which included a psychosocial questionnaire including items regarding perceptions of neighborhood disorder. The other half of the sample completed the EFTF interview at the next wave of data collection in 2008. Half-sample-based variables were combined in the present study for complete data on perceived neighborhood disorder. The present study uses the most recent 2016–2018 waves of data from HRS. Additionally, in 2016, HRS participants provided a venous blood sample that allowed for the assay of several biomarkers of physiological functioning, including fasting glucose levels which serve as an indicator of potential prediabetes and diabetes. Participant health records were linked via geographic identifiers to a Contextual Data Resource (CDR) (43), including data from the American Community Survey, which enables investigation of health in the context of broad neighborhood environments. All participants signed consent forms prior to any data collection and all research procedures were approved by the University of Michigan’s Institutional Review Board.

The analytic sample in the present study included those who reported whether they had type 2 diabetes and complete data on all analytic variables. Individuals who could not be categorized as non-Hispanic White, non-Hispanic Black, or Hispanic were categorized as non-Hispanic ‘Others.’ Given that this subgroup was small and racially/ethnically diverse, precluding meaningful comparison, those individuals were not included in the present analyses, resulting in an analytic sample of 10,419 respondents.

### 2.2 Measures

#### 2.2.1 Type 2 diabetes

During the 2016 core interview, participants answered the question, “Has a doctor ever told you that you have diabetes or high blood sugar?” Respondents who reported that they currently have or have had diabetes were coded as (1) and coded as (0) if they did not have or never had diabetes. A dichotomous fasting glucose variable was constructed that categorized individuals as 0 (below the clinical cut point of 125 mg/dL) or 1 (at or above 125 mg/dL) to be used in a sensitivity analysis.

#### 2.2.2 Perceived neighborhood disorder

During the 2016 Psychosocial and Lifestyle Leave-Behind Questionnaire, participants answered four questions assessing social and physical disorder in their neighborhoods (21). These questions asked about the degree to which participants perceived vandalism, trash, and vacant buildings in their neighborhoods as well as how safe people likely feel walking alone in their neighborhoods. Participants responded to these questions using a 7-point Likert-type scale, and these responses were then averaged with a higher score representing greater perceived neighborhood disorder (Cronbach’s α = 0.84).

#### 2.2.3 Race/ethnicity

The 2016 HRS Tracker File contained participant racial/ethnic status of each participant. Race/ethnicity categories included Non-Hispanic Whites (1), Non-Hispanic Blacks (2), and Hispanics (3).

#### 2.2.4 Covariates

Several variables known to correlate with neighborhood characteristics, type 2 diabetes, or both were included as covariates in the present analyses. Participants’ highest levels of education, sex, and age were drawn from the 2016 Tracker File Version 3.0. Education was coded using the following categories: 0 = No degree, 1 = GED, 2 = High school diploma, 3 = Two-year college degree, 4 = Four-year college degree, 5 = master’s degree, and 6 = Professional degree (Ph.D., M.D., J.D.). Sex was coded as 0 = male and 1 = female. Age was coded in years. Derived from the RAND-contribution 2018 V1 file, total household wealth in 2016 was calculated by summing all sources of income from both participant and spouse (e.g., earnings, social security payments, Medicare Part B, pension and retirement, interest, rents, educational assistance, alimony), and subtracting from this all sources of debt (e.g., mortgages from primary and secondary homes, other home loans, and sources of debt not asked) (44). Although for descriptive purposes the average and standard error of this household wealth variable is listed in Table 1 in its original unit of measurement, for ease of interpretation, a standardized version of this variable was used in all analytic models so that coefficients could be interpreted as a change in type 2 diabetes risk for a standard deviation-increase in household wealth.

**Table 1:** Descriptive statistics, full sample and stratified by race/ethnicity (mean [sd]).

	Full sample (*n* = 10,419)	Non-Hispanic Whites (*n* = 7,094)	Non-Hispanic Blacks (*n* = 1,910)	Hispanics (*n* = 1,415)
Type 2 diabetes risk	22%	19%	33%	36%
Perceived neighborhood disorder	2.40 (0.02)	2.25 (0.02)	3.27 (0.05)	3.01 (0.06)
**Educational degree** ^**a**^				
GED	4.60%	4.31%	6.42%	5.84%
High school diploma	44.65%	45.16%	47.63%	36.39%
2-year degree	7.63%	7.88%	7.74%	4.97%
4-year degree	19.44%	21.10%	13.72%	7.97%
Master’s degree	11.32%	12.33%	5.98%	6.20%
Professional degree	3.49%	3.97%	0.96%	1.06%
Women^b^	56%	55%	61%	54%
Age (years)	66.10 (0.11)	66.48 (0.13)	64.87 (0.26)	63.47 (0.30)
Household wealth ($)	504 K (17 K)	571 K (19 K)	117 K (8 K)	203 K (27 K)
Concentrated disadvantage	−0.21 (0.01)	−0.36 (0.01)	0.70 (0.03)	0.42 (0.04)
Population density	3,857 (111.58)	2,994 (116.88)	7,176 (428.48)	9,441 (638.76)
Racial/ethnic diversity	0.34 (0.00)	0.33 (0.00)	0.43 (0.01)	0.40 (0.01)

sd, standard deviation. Household wealth is rounded to the nearest $100,000 ($10,000). ^a^Compared to no degree. ^b^Compared to men.

Three census-tract-level variables from the ACS 2012–2016 five-year estimates were included from the HRS CDR, version 2.0 (36, 43). First, concentrated disadvantage was constructed by averaging three standardized variables: the proportion of households in which household income falls at or below the federal poverty threshold, the proportion of households for which the head of household is unemployed, and the proportion of female-headed households. Second, population density was defined as population per square mile. Third, racial/ethnic diversity was constructed by subtracting from the total population of the census tract the proportions of the following racial/ethnic groups: non-Hispanic White, non-Hispanic Black, non-Hispanic Asian, non-Hispanic Other, and Hispanic (45).

### 2.3 Statistical analysis

Participants are recruited to HRS using a complex survey design. A thorough description of the survey design is provided elsewhere (46). Given that the primary outcome of the current analyses was self-reported diabetes status, which was a question asked of all participants as part of the 2016 core interview, the 2016 household analysis weight was used. This weight variable compensates for unequal selection probabilities due to oversampling of Hispanics, Blacks, and households in Florida, adjusts for response rate differences across race groups and geographic areas, and further adjusts for the subsampling of households located in the most dangerous areas in the US [see Heeringa and Connor (46) for more details]. Prior to formal analysis, the data set was structured in Stata 16 using the svyset command which applies the above weight variable to the analytic results and organizes the respondents into 80 stratum based on the above sampling design. Weighted logistic regressions were conducted in Stata 16 using the svy: suite of commands. These models examined the hypothesized main and interaction effects of perceived neighborhood disorder and racial/ethnic status on self-reported type 2 diabetes status. Although there is a relatively smaller sample of participants in HRS with biological data relative to those with a self-report of diabetes status, we nevertheless found it important to compare results of the above model investigating self-reported diabetes with a separate model investigating glucose levels above the clinical cut point of 125 mg/dL to determine the degree of consistency in the results. In a sensitivity analysis, weighted logistic regressions were conducted to predict the likelihood of having glucose levels above the clinical cut point of 125 mg/dL by race/ethnicity, perceived neighborhood disorder, and their interaction. All models were adjusted for highest educational degree, sex, age, household wealth, as well as census tract-level concentrated disadvantage, population density, and racial/ethnic diversity.

## 3 Results

### 3.1 Participant description

A description of the weighted sample and samples stratified by race/ethnicity can be found in Table 1. The full sample was 65% non-Hispanic White, 18% Black/African-American, and 13% Hispanic. About 22% of the sample reported having type 2 diabetes, and participants reported somewhat low levels of perceived neighborhood disorder. Roughly 34% of the sample earned a four-year degree or greater compared to those with a two-year degree or less.

### 3.2 Race/ethnicity, perceived neighborhood disorder, and type 2 diabetes

Hispanics reported higher type 2 diabetes prevalence than non-Hispanic Blacks or non-Hispanic Whites. Non-Hispanic Blacks reported the highest perceived neighborhood disorder, followed by Hispanics and non-Hispanic Whites. A greater proportion of non-Hispanic Blacks earned a two-year degree or less when compared to non-Hispanic Whites or Hispanics. Non-Hispanic Whites represented the largest proportion of participants with four-year degrees or greater, followed by non-Hispanic Blacks and Hispanics. Participants in the different racial/ethnic groups were of similar age, and there were more women than men in all the racial/ethnic groups. Non-Hispanic Whites had greater household wealth compared to Hispanics and non-Hispanic Blacks. The neighborhoods represented by non-Hispanic Whites were, on average, less economically disadvantaged, less densely populated, and less racially-ethnically diverse than those of non-Hispanic Blacks or Hispanics.

**Figure 1:**
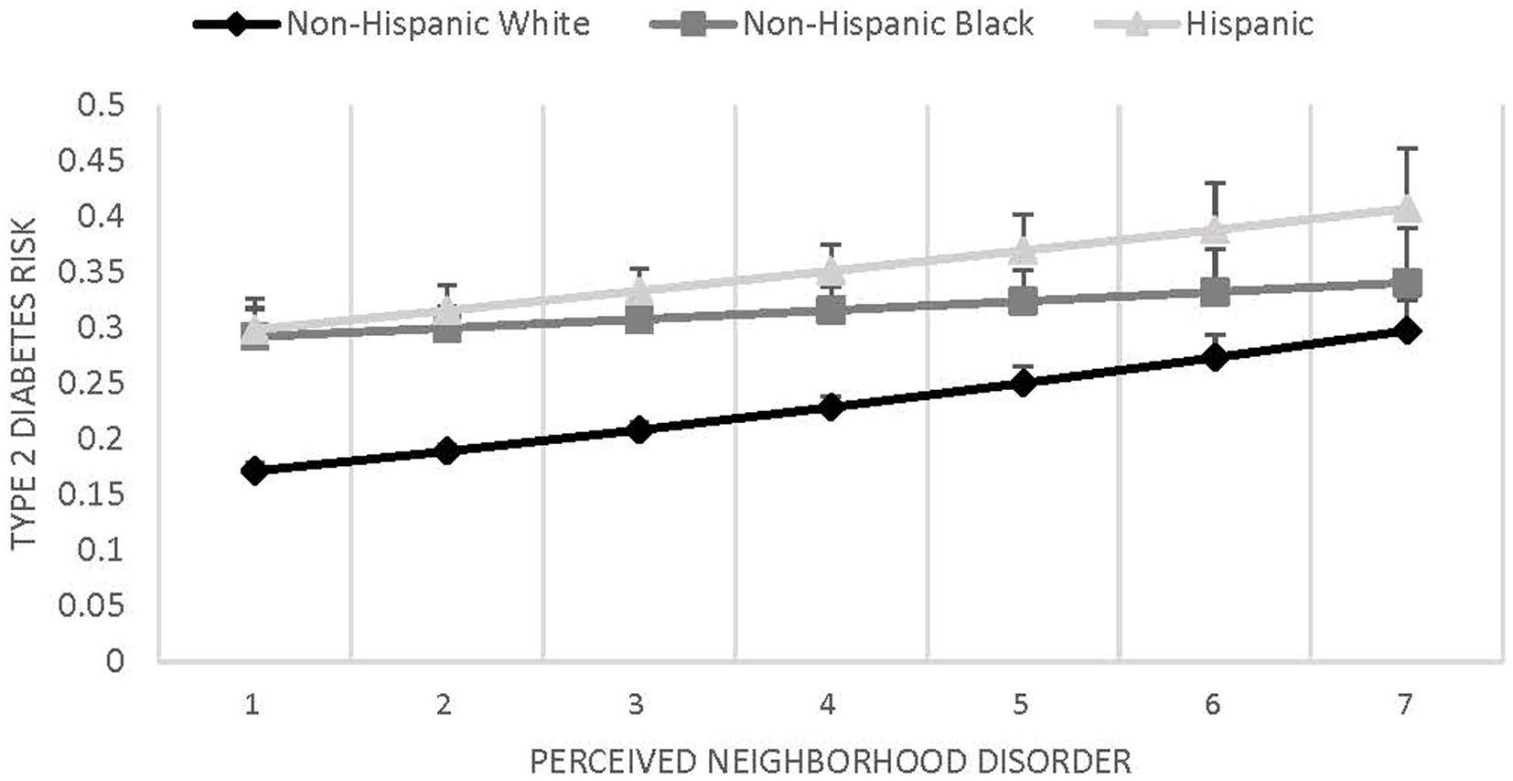
Perceived neighborhood disorder × race/ethnicity on type 2 diabetes.

Results of the statistical models evaluating type 2 diabetes prevalence in relation to race/ethnicity, perceived neighborhood disorder, and their interaction are shown in Table 2. Results of Model 1 indicate that people who perceived their neighborhoods as more disordered had greater risk of type 2 diabetes (coef. = 1.11, *SE* = 0.03, *p* < 0.001). Non-Hispanic Blacks (coef. = 1.69, *SE* = 0.16, *p* < 0.001) and Hispanics (coef. = 1.95, *SE* = 0.20, *p* < 0.001) had greater risk of type 2 diabetes compared to non-Hispanic Whites. Model 2 assessed the potential interaction between race/ethnicity and neighborhood disorder on type 2 diabetes. The interaction was not significant (see Figure 1), suggesting that the relationship between perceived neighborhood disorder and type 2 diabetes did not significantly differ between non-Hispanic Whites and either non-Hispanic Blacks (coef. = 0.92, *SE* = 0.05, *p =* 0.141) or Hispanics (coef. = 0.96, *SE* = 0.06, *p =* 0.490). To address potential common source bias, the above analyses were repeated with an alternative outcome variable that dichotomized glucose values to below or at and above the clinical cut-point. These analyses revealed a similar pattern of results. Given the reduced number of participants with biological data, coupled with the moderate and statistically significant correlation between the dichotomous glucose variable and self-reported diabetes status (corr. = 0.51, *p* < 0.001), we report only the original results with self-reported type 2 diabetes status.

**Table 2:** Weighted logistic regression predicting type 2 diabetes risk (*n* = 10,419).

	Model 1	Model 2
Perceived neighborhood disorder	1.11^***^ (0.03)	1.13^***^ (0.03)
Race/ethnicity^a^		
Non-Hispanic Black	1.69^***^ (0.16)	2.20^***^ (0.41)
Hispanic	1.95^***^ (0.20)	2.18^***^ (0.43)
Race/ethnicity × perceived neighborhood disorder		
Non-Hispanic Black		0.92 (0.05)
Hispanic		0.96 (0.06)
Education^b^		
GED	1.04 (0.16)	1.03 (0.16)
High school diploma	0.90 (0.08)	0.90 (0.08)
2 year degree	0.80 (0.12)	0.79 (0.12)
4 year degree	0.62^***^(0.08)	0.62^***^ (0.08)
Master’s degree	0.73^*^ (0.11)	0.73^*^ (0.11)
Professional degree	0.78 (0.17)	0.79 (0.17)
Household wealth	0.79^**^ (0.06)	0.79^**^ (0.06)
Age	1.02^***^ (0.00)	1.02^***^ (0.00)
Sex^c^	0.75^***^ (0.05)	0.75^***^ (0.05)
Concentrated disadvantage	1.05 (0.05)	1.05 (0.05)
Population density	1.00 (0.00)	1.00 (0.00)
Racial/ethnic diversity	0.82 (0.14)	0.80 (0.13)

^*^p < 0.05; ^**^p < 0.01; ^***^p < 0.001. ^a^Compared to Non-Hispanic White. ^b^Compared to no degree. ^c^Compared to men.

## 4 Discussion

There are well-established racial/ethnic disparities in health outcomes including type 2 diabetes. This particular health outcome is rooted in many social, behavioral, and environmental risk factors (47, 48). Importantly, there is an unequal distribution of these risk factors across racial/ethnic groups (49, 50). To our knowledge, ours is among the first research studies to investigate racial/ethnic differences in the relationship between perceived neighborhood disorder and type 2 diabetes risk with a representative older US sample. Results suggested that type 2 diabetes risk was elevated among those reporting more neighborhood social and physical disorder regardless of racial/ethnic status. As such, these findings suggest that neighborhood disorder is an important target for community-level interventions aimed at reducing the prevalence and incidence of type 2 diabetes and these interventions may be particularly beneficial for members of marginalized racial/ethnic groups who are most likely to live in disordered neighborhoods.

### 4.1 Our findings in context: the importance of creating cleaner and safer communities

Theory and previous research suggest racial/ethnic differences in the associations between neighborhood disorder and various psychosocial outcomes, including mastery (39) and personal control (40). This research study enriches the dialog about how neighborhood disorder impacts a physical health outcome, namely type 2 diabetes. While Gilster (39) and Kim and Conley (40) posited that racial/ethnic groups may be differentially impacted by neighborhood disorder, we observed no difference across racial/ethnic groups regarding the link between perceived neighborhood disorder and type 2 diabetes. This pattern of results demonstrates neither support for the Status Discord Theory (40) nor the Compound Risk Theory (39), suggesting that physical health outcomes may not be fully understood through the lens of existing neighborhood disorder theories.

More research should be performed to identify mechanisms explaining the neighborhood disorder-health associations. Such investigations would inform the development of community-level interventions targeting neighborhood disorder. Results of the present study suggest that such intervention may contribute to reducing type 2 diabetes prevalence and racial/ethnic disparities therein. Some efforts, such as the Racial and Ethnic Approaches to Community Health (REACH) program administered by the CDC in 1999, already exist. The REACH program provides funding and resources to local organizations and institutions in various US states. These funds are provided for the creation of initiatives that combat disproportionate prevalence of diabetes among marginalized racial/ethnic groups (51). Investment in community resources that encourage more physical and social engagement may support the maintenance of healthy lifestyles and thus improve community-level health, particularly for those who are most embedded in disordered neighborhoods.

### 4.2 Limitations and future directions

This study made several important contributions, but they are not without limitations. Despite the national representativeness of the HRS sample, the relatively larger sample of non-Hispanic Whites in HRS creates differences in statistical power to detect statistically significant links between perceived neighborhood disorder and type 2 diabetes across these racial/ethnic groups. Neighborhood effects are generally small (52), and this is likely true for perceived neighborhood disorder, so it may be the case that the larger sample of non-Hispanic Whites in HRS was better powered to detect its association with health in the present study. Although not shown in our analysis table, we conducted unweighted analyses and observed a statistically significant racial/ethnic difference in the perceived neighborhood disorder-type 2 diabetes association; higher perceived neighborhood disorder was related to a greater likelihood of having type 2 diabetes, but only among Hispanics and non-Hispanic Whites. This trend was evident in Figure 1, but was nevertheless not statistically significant in weighted analyses, perhaps due to the fact that HRS weights compensate for the oversampling of non-Hispanic Blacks in the United States.

A few other limitations are worth noting. Data collection efforts should be made to sample participants living in neighborhoods with a wider range of disorder, as the measure of perceived neighborhood disorder in the present study was somewhat truncated and represented neighborhoods perceived as having lower levels of disorder. HRS does not provide a means to reclassify non-Hispanic “Others” into more meaningful categories, and as such, this group was excluded from the current analyses. The outcome and neighborhood predictor were both self-reported in this study which may have introduced common source bias. However, this final concern is attenuated by research indicating that self-reports of neighborhood disorder and objectively-assessed levels of neighborhood disorder are highly correlated (53). Moreover, our reported results were compared to a model that exchanged self-reported diabetes for measured fasting glucose levels. There were no substantive differences in the results of these two models. Nevertheless, the sample of HRS participants with measured glucose levels was much smaller when compared to the sample with self-reported diabetes information, and as such, results of the model with self-reported data were used in the present research. A related concern is that the item asking participants about their diabetes status, “Has a doctor ever told you that you have diabetes or high blood sugar?” may also include individuals with prediabetes. Future studies should test these questions using larger samples with biological data.

## Data availability statement

Publicly available datasets were analyzed in this study. This data can be found at: https://hrs.isr.umich.edu/about.

## Ethics statement

The studies involving humans were approved by Chapman University Institutional Review Board. The studies were conducted in accordance with the local legislation and institutional requirements. Written informed consent for participation was not required from the participants or the participants’ legal guardians/next of kin because This study involved exclusive secondary analysis of existing data.

## Author contributions

MY: Conceptualization, Formal Analysis, Writing – original draft. AV: Conceptualization, Formal Analysis, Writing – original draft. BC: Conceptualization, Supervision, Writing – review & editing. JR: Conceptualization, Formal Analysis, Supervision, Writing – review & editing.
